# Curcumin Induces EGFR Degradation in Lung Adenocarcinoma and Modulates p38 Activation in Intestine: The Versatile Adjuvant for Gefitinib Therapy

**DOI:** 10.1371/journal.pone.0023756

**Published:** 2011-08-17

**Authors:** Jen-Yi Lee, Yee-Ming Lee, Gee-Chen Chang, Sung-Liang Yu, Wan-Yu Hsieh, Jeremy J. W. Chen, Huei-Wen Chen, Pan-Chyr Yang

**Affiliations:** 1 Department and Institute of Pharmacology, School of Medicine, National Yang-Ming University, Taipei, Taiwan; 2 Graduate Institute of Toxicology, College of Medicine, National Taiwan University, Taipei, Taiwan; 3 Division of Chest Medicine, Department of Internal Medicine, Taichung Veterans General Hospital, Taichung, Taiwan; 4 Institute of Biomedical Science, College of Life Sciences, National Chung-Hsing University, Taichung, Taiwan; 5 Department of Clinical Laboratory Sciences and Medical Biotechnology, College of Medicine, National Taiwan University, Taipei, Taiwan; 6 Department of Internal Medicine, National Taiwan University Hospital and National Taiwan University Medical College, Taipei, Taiwan; Roswell Park Cancer Institute, United States of America

## Abstract

**Background:**

Non-small cell lung cancer (NSCLC) patients with L858R or exon 19 deletion mutations in epidermal growth factor receptor (EGFR) have good responses to the tyrosine kinase inhibitor (TKI), gefitinib. However, patients with wild-type EGFR and acquired mutation in EGFR T790M are resistant to gefitinib treatment. Here, we showed that curcumin can improve the efficiency of gefitinib in the resistant NSCLC cells both *in vitro* and *in vivo* models.

**Methods/Principal Findings:**

After screening 598 herbal and natural compounds, we found curcumin could inhibit cell proliferation in different gefitinib-resistant NSCLC cell lines; concentration-dependently down-regulate EGFR phosphorylation through promoting EGFR degradation in NSCLC cell lines with wild-type EGFR or T790M EGFR. In addition, the anti-tumor activity of gefitinib was potentiated via curcumin through blocking EGFR activation and inducing apoptosis in gefitinib-resistant NSCLC cell lines; also the combined treatment with curcumin and gefitinib exhibited significant inhibition in the CL1-5, A549 and H1975 xenografts tumor growth in SCID mice through reducing EGFR, c-MET, cyclin D1 expression, and inducing apoptosis activation through caspases-8, 9 and PARP. Interestingly, we observed that the combined treatment group represented better survival rate and less intestinal mucosal damage compare to gefitinib-alone therapy. We showed that curcumin attenuated the gefitinib-induced cell proliferation inhibition and apoptosis through altering p38 mitogen-activated protein kinase (MAPK) activation in intestinal epithelia cell.

**Conclusions/Significance:**

Curcumin potentiates antitumor activity of gefitinib in cell lines and xenograft mice model of NSCLC through inhibition of proliferation, EGFR phosphorylation, and induction EGFR ubiquitination and apoptosis. In addition, curcumin attenuates gefitinib-induced gastrointestinal adverse effects via altering p38 activation. These findings provide a novel treatment strategy that curcumin as an adjuvant to increase the spectrum of the usage of gefitinib and overcome the gefitinib inefficiency in NSCLC patients.

## Introduction

Lung cancer is the leading cause of cancer death in the United States and worldwide [1], [2]. Approximately 85% of lung cancer patients belong to the non-small-cell lung cancer (NSCLC) group with a poor prognosis [3]. Previous study showed that the epidermal growth factor receptor (HER1/EGFR) overexpression rate is 40-80% in NSCLC and plays the key role in tumorigenesis [4]. In addition, EGFR-driven cell signaling contributes to the disease progression and cancer malignancy [5]. These offer an effective therapeutic target to develop agents for NSCLC [6].

Gefitinib, EGFR tyrosine kinase inhibitor (TKI), was the first selective small molecular agent showed the effective activity in blocking EGFR phosphorylation and downstream signaling and approved for NSCLC treatment [7], [8]. Previous multi-institutional clinical trial studies indicated the response to gefitinib is better in Asian patients compared to Caucasian patients and that women who are non-smokers and have adenocarcinoma are the most likely to benefit the most [9], [10]. In addition, recent studies showed the pharmacogenomic issues in *EGFR* determine the limitation of gefitinib in clinical applications: Activating mutations of the in-frame deletions (ΔE746-A750) in exon 19 and the L858R point mutation in exon 21 of the EGFR tyrosine kinase domain in NSCLC are highly correlated with gefitinib sensitivity [11], [12], [13]. However, the activating mutations rate has been found in range from 10% to 15% in Caucasians and from 30% to 40% in Asians among the NSCLC patients [14], [15]. In addition, acquired resistance caused by a second site substitution, T790M in exon 20, results in poorly gefitinib activity [16], [17]. Thus, it is important and urgent to develop new agents or pinpoint novel approaches for improving the anti-tumor effects of gefitinib in NSCLC patients.

By screening the 598 herbal and natural compounds containing the range of alkaloids, sesquiterpenes, diterpenes, pentacyclic triterpenes, sterols and many other diverse representatives purchased from Sigma and ChromaDex to the different gefitinib-resistant NSCLC cell lines through cell proliferation assay, we tested one of the hits curcumin (diferuloylmethane) as the candidate may be a potential agent to accomplish our goal. We have previously shown that curcumin could inhibit cell cycle progression, induce cell apoptosis and anti-metastasis by regulating various mechanisms in different cell types [18], [19], [20]. Recent studies indicated that curcumin was able to down-regulate EGFR and HER2/neu protein kinase activity which inhibits the cancer cells growth [21], [22], [23]. In addition, curcumin has been reported to suppress the growth of human colon cancer-derived Moser cells by reducing *EGFR* genes expression [24]. Curcumin also down-regulates EGFR signaling in prostate cancer cells by modulating levels of EGFR protein showing that curcumin inhibits the intrinsic EGFR tyrosine kinase activity and suppress ligand-induced activation of EGFR [25]. Furthermore, clinical trials have indicated that no dose-limiting toxicity when curcumin is administered at doses up to 10 g/day and the serum concentration of curcumin was 1.77±1.87 µM at the dose of 8 g/day [26]. Thus, curcumin may have enormous potential and relative cheap and safe in cancer therapy combined with other treatments to NSCLC.

Although the anticancer mechanisms of curcumin and the antineoplastic activities of curcumin combined with several clinical used antitumor drugs have been documented, the potential effects of curcumin combine with gefitinib in NSCLC have not been evaluated. In the present study, we determine curcumin as a potential agent by the high-throughput drug screening and examine that curcumin suppress EGFR activation was resulted from accelerating EGFR protein degradation. We next investigate the effectiveness of curcumin on anti-tumor effects of gefitinib in NSCLC *in vitro* and *in vivo*. We reveal that curcumin enhances the anti-tumor effects of gefitinib in various resistant NSCLC cancer cell lines by modulating the EGFR protein kinase activity which leads to reduce the cancer cells growth ability and promotes cell apoptosis *in vitro*. Besides, curcumin also significantly potentiates the anti-tumor properties of gefitinib in gefitinib-resistant cancer cells bearing subcutaneously tumors in SCID mice. Interestingly, we observe that curcumin significantly reduces the gastrointestinal (GI) adverse effect of gefitinib in xenografts model. We find that curcumin significantly attenuates the gefitinib-induced GI damage and resulted in a better survival rate with less damage to the villi might through modulating p38 activation.

## Results

### Curcumin inhibits cell proliferation in gefitinib-resistant lung adenocarcinoma cells and induces endogenous EGFR protein degradation

**Figure 1:**
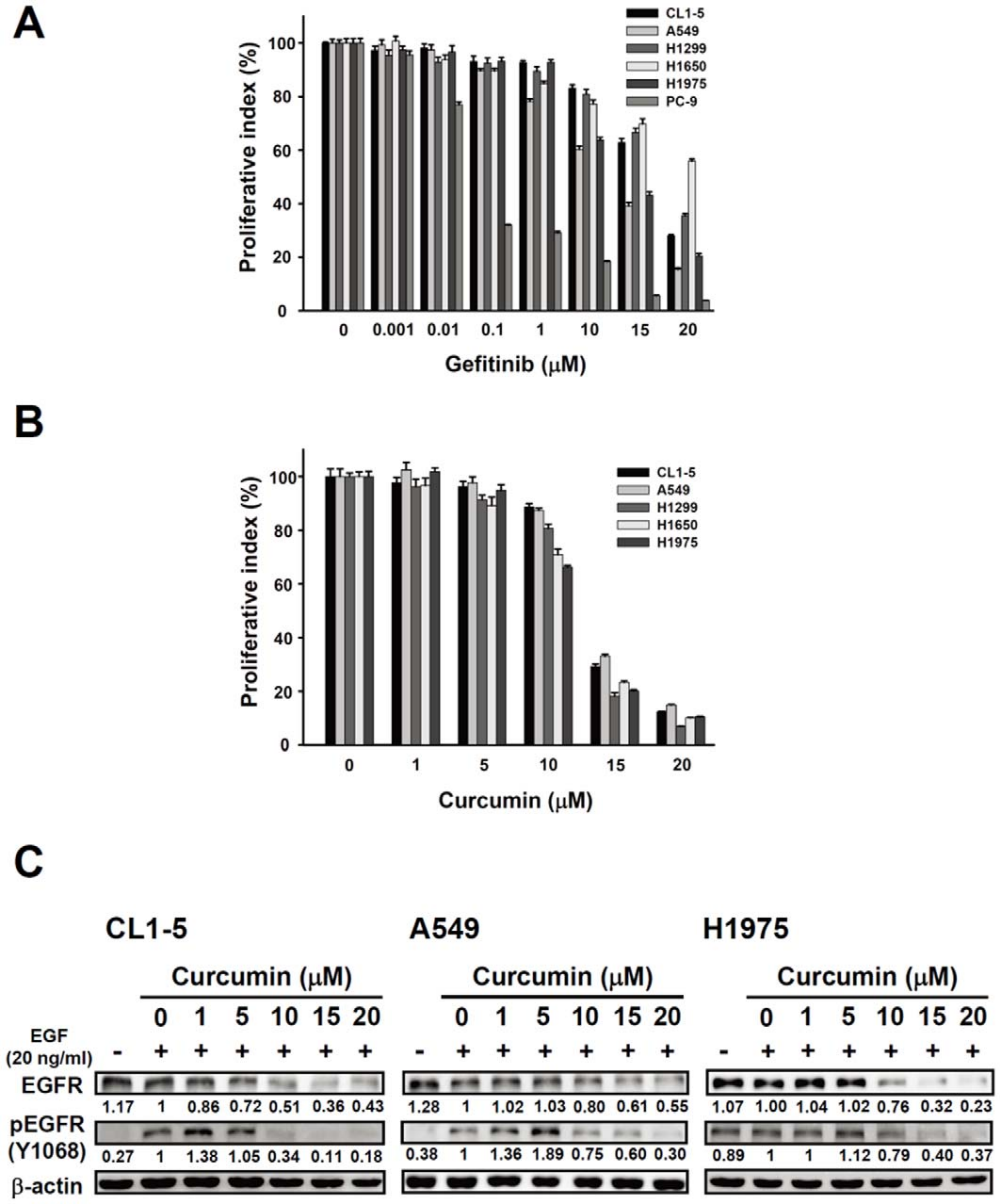
Curcumin inhibits cell proliferation and down-regulates EGFR activation in gefitinib-resistant NSCLC cell lines. (A) MTT assay results showed susceptibility of gefitinib in all six lung adenocarcinoma cell lines. *Columns*, mean (n = 6); *bars*, SEM. Data are representative of two independent experiments. (B) MTT assay analysis showed curcumin harbored dose-dependent suppression of cell proliferation in five gefitinib-resistant NSCLC cell lines. *Columns*, mean (n = 6); *bars*, SEM. Data are representative of two independent experiments. (C) Western blotting analysis showed curcumin decrease the EGF (20 ng/ml)-induced EGFR and pEGFR, expression in CL1-5, A549 and H1975 cells in a dose-dependent manner (1–20 µM). β-actin used as the internal control and the numbers below the row of the EGFR and pEGFR indicate the densitometric values normalized with the relative β-actin value.

To screen the potential hits which can overcome the gefitinib resistance, several gefitinib resistance NSCLC cell lines, including CL1-5 (EGFR^wt^), A549 (EGFR^wt^), H1299 (EGFR^wt^), H1650 (EGFR^19del^) and H1975 (EGFR^L858R+T790M^) were used, which showed an overall pattern of increased resistance when incubated with gefitinib for 72 h (IC_50_ = 15–20 µM for CL1-5, A549, H1299 and H1975, H1650 is higher than 20 µM), compared with the gefitinib-sensitive cell lines, PC-9 (EGFR^19del^)(IC_50_ = 30 nM) (Figure 1A), Screening 598 herbal and natural compounds; we obtained 26 potential candidates such as curcumin, emetine, shikonin and tomatine (data not shown). Further validation showed curcumin significantly inhibits cell proliferation with no difference in EGFR status of these gefitinib-resistant lung adenocarcinoma cells in a concentration-dependent manner (Figure 1B).

**Figure 2:**
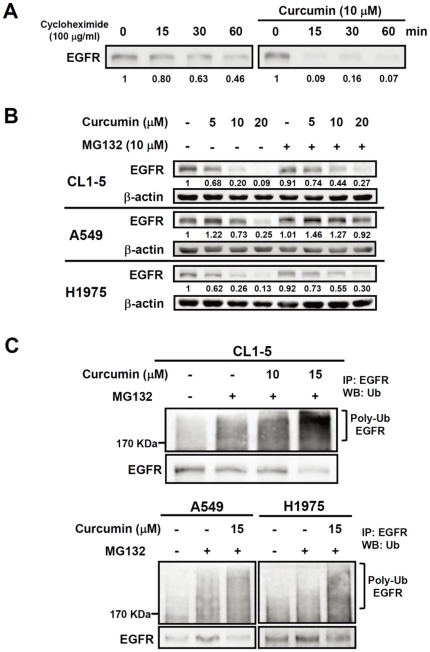
Curcumin potentially modulates EGFR expression by accelerating protein degradation in gefitinib-resistant NSCLC cell lines. (A) Analysis the half-life of EGFR protein level in 100 µg/ml cycloheximide treated CL1-5 cells with or without 10 µM curcumin by Western blotting. (B) Western blotting analysis revealed that MG132 recover the EGFR level of the curcumin-affected EGFR protein depletion. β-actin used as the internal control and the numbers below the row of the EGFR indicate the densitometric values normalized with the relative β-actin value. (C) Detection of ubiquitin (Ub) levels by immunoprecipitation of EGFR in CL1-5, A549 and H1975 cells showed the elevation of poly-Ub expressions in the presence of curcumin (10–15 µM). Ub and EGFR levels were measured by Western blotting.

In terms of the EGFR is highly correlated with the tumor progression in lung cancer [4], [5], wild-type EGFR is the main population among the NSCLC cases, and EGFR T790M results in TKIs resistance [14], [15], [16], [17]. The effects of curcumin on EGFR activation in CL1-5, A549 and H1975 cells were examined. Our results indicated that the starved cells pre-treated with curcumin (0–20 µM) and then stimulated with 20 ng/ml EGF for 3 h showing the concentration-dependently reduced phospho-EGFR protein levels in these NSCLC cell lines; interestingly, the endogenous EGFR were also dramatically depleted (Figure 1C). To investigate the possible mechanism underlying curcumin-induced EGFR depletion, we examined whether curcumin accelerates EGFR degradation and results in EGFR protein destabilization. Treating CL1-5 cells with the protein synthesis inhibitor, cycloheximide (100 µg/ml), the EGFR protein level decreased slowly; whereas, curcumin can quickly induce EGFR depletion to 10% in 15 min (Figure 2A). Interestingly, the curcumin-induced EGFR degradation in CL1-5, A549, and H1975 cells all in the concentration-dependent manner, and at least, in part, through proteasome-dependently; whereas, Figure 1D showed that the proteasome inhibitor, MG132 (10 µM), can partially recover the curcumin-induced EGFR degradation (Figure 2B). Furthermore, the immuno-precipitation data showed that curcumin may induce EGFR protein degradation through ubiquitination of wild-type EGFR (CL1-5 and A549 cells) and also in the mutated EGFR with T790M (H1975 cells) (Figure 2C). These results indicated that curcumin-induced EGFR degradation may go through accelerating ubiquitin-proteasome ability in the gefitinib-resistant cells.

### Curcumin potentiates the anti-cancer effects of gefitinib in human lung adenocarcinoma cells *in vitro*

**Figure 3:**
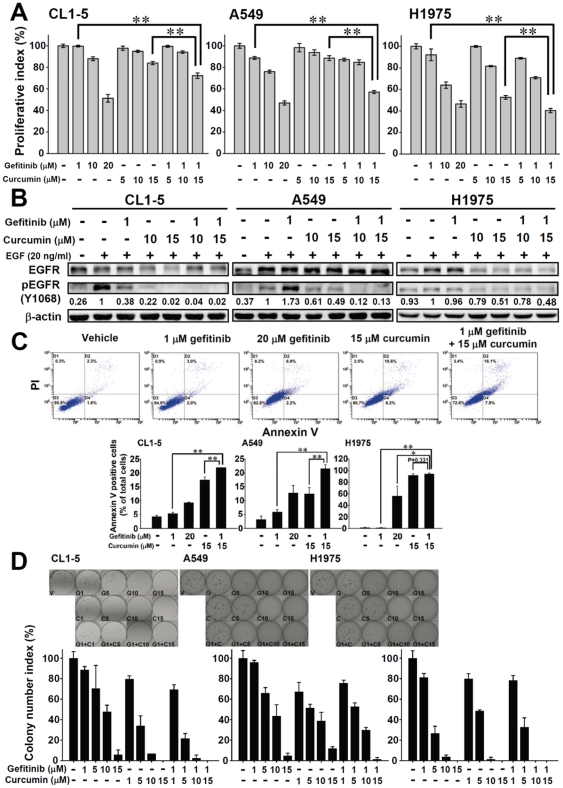
Curcumin potentiates antitumor abilities of gefitinib in cell proliferation, EGFR activation, apoptotic ability, and colony formation of gefitinib in gefitinib-resistant in NSCLC *in vitro*. (A) MTT assay results showed curcumin enhanced the anti-proliferative effect of gefitinib in CL1-5, A549 and H1975 gefitinib-resistant lung adenocarcinoma cell lines. *Columns*, mean (n = 6); *bars*, SEM. **, *P*<0.01. Data are representative of two independent experiments. (B) Western blotting analysis showed curcumin improve the blockage of EGF (20 ng/ml)-induced EGFR and pEGFR expression of gefitinib in CL1-5, A549 and H1975 cell lines. β-actin used as the internal control and the numbers below the row of the pEGFR indicate the densitometric values normalized with the relative β-actin value. (C) Annexin V-FITC apoptosis assay results showed curcumin enhance the apoptotic effect of gefitinib, CL1-5 cell was treated agents as indicating. The *x* axis is Annexin-V-FITC, and the *y* axis is PI (propidium iodide) for all graphs represented; CL1-5, A549 and H1975 cells undergoing apoptosis were statistically counted the Annexin-V^+^ and PI^-^ levels of total cells in the lower right quadrant. *Columns*, mean (n = 3); *bars*, SD. *, *P*<0.05, **, *P*<0.01 and *P* = 0.331 (15 µM curcumin versus 1 µM gefitinib plus 15 µM curcumin). Data are representative of triplicate independent experiments. (D) Colony formation assay analysis represented curcumin enhance the colony inhibitory ability of gefitinib in CL1-5, A549 and H1975 cells. Cells cell was treated agents as indicating; V: vehicle control, G1, G5, G10, G15, C1, C5, C10, C15, G1+C1, G1+C5, G1+C10 and G1+C15: G and C indicated gefitinib and curcumin, and number showed the concentrations (µM) of the agents, respectively; Colony formation index (%) was expressed as the percent of cells number in the each groups compared to the vehicle control. Colonies were counted after 2 weeks of the experiment using crystal violet staining. *Columns*, mean (n = 3); *bars*, SD. Data are representative of two independent experiments.

Curcumin showed potential effects on inhibiting cancer cell proliferations and can induce EGFR degradation in gefitinib-resistant NSCLC cell lines. Previous study indicated that curcumin has been shown to have therapeutic effects against cancer and may play a ideal agents for cancer therapy [27]. We hypothesized that curcumin may enhance the anti-cancer effects of gefitinib on these resistant cell lines; including CL1-5, A549 and H1975 cell lines (the IC_50_ of these cell lines to gefitinib are 10–15 µM). Curcumin exhibited to sensitize the anti-proliferative ability of gefitinib of all three gefitinib-resistant cell lines, and the combination of curcumin and gefitinib was equivalent in cell viability to the high-dose gefitinib (Figure 3A). Combination index (CI) isobologram was also calculated as descript in “Material and Methods” and showed that combination treatment with curcumin and gefitinib synergistically in CL1-5 (EGFR^wt^)(CI = 0.45), A549 (EGFR^wt^)(CI = 0.41), H1299 (EGFR^wt^)(CI = 0.66), and H1650 (EGFR^19del^)(CI = 0.83); whereas, approximately addictive in H1975 (EGFR^L858R+T790M^) cell line (CI = 1.02). Furthermore, combination treatments with curcumin (10–15 µM) and gefitinib (1 µM) can almost complete blockage the EGFR activation in the three gefitinib-resistant cells (CL1-5, A549 and H1975 cell lines) (Figure 3B).

To examine whether curcumin can increase gefitinib-induced cell apoptosis in the gefitinib-resistant cell lines, flow cytometry assay with propidium iodide/annexin-V staining was used, and the results indicated that 1 µM gefitinib combined with 15 µM curcumin induced more apoptotic cells than 20 µM gefitinib in CL1-5 and A549 cells (Figure 3C). Curcumin showed most effectively on H1975 cells; since curcumin can induce around 90% of cell apoptosis in H1975 cell with or without gefitinib.

We next examined whether curcumin enhances the anti-tumorigenicity of gefitinib in the gefitinib-resistant cell lines using a colony formation assay. The results showed that combination of 1 to 15 µM curcumin and 1 µM gefitinib significantly inhibited colony formation by CL1-5, A549 and H1975 compared to the drug-free control and either gefitinib (1–15 µM) or curcumin (1–15 µM) treatment alone, respectively (Figure 3D).

### Curcumin enhances the antitumor properties of gefitinib in human lung adenocarcinoma cells xenografts *in vivo*

**Figure 4:**
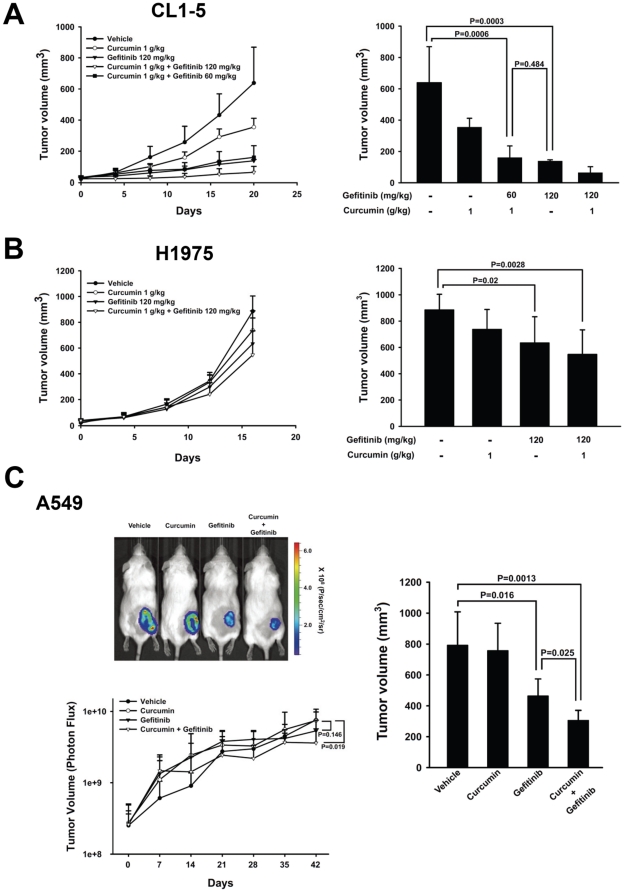
Curcumin sensitizes the effect of gefitinib in inhibiting the growth of gefitinib-resistant NSCLC in SCID mice. (A) *left*, 10^6^ CL1-5 cells were implanted s.c. in SCID mice and tumor volumes were monitored over time. *Points,* mean (n = 6); *bars*, SD; *right*, tumor volume in CL1-5 xenograft were measured on the last day of the experiment using vernier calipers. *Columns*, mean (n = 6); *bars*, SD. *P* = 0.0003 (control versus gefitinib), 0.0006 (control versus 1 g/kg and 60 mg/kg gefitinib) and 0.484 (1 g/kg and 60 mg/kg gefitinib versus gefitinib). (B) *left*, 10^6^ A549 xenograft study processed as well as the methods in CL1-5 xenograft. *Points*, mean (n = 6); *bars*, SD; *right*, tumor volume in A549 xenograft were measured on the last day of the experiment using vernier calipers. *Columns*, mean (n = 6); *bars*, SD. *P* = 0.0028 (control versus combination), 0.02 (control versus gefitinib). (C) *left*, bioluminescence IVIS images of A549 xenograft mice were visualized in anesthetized animals after i.p. inoculation of D-luciferin. The photon flux were measured per second and depicted as the tumor volume at the indicated time using IVIS imaging. *Points*, mean (n = 6); *bars*, SD. *P* = 0.019 (control versus combination), 0.146 (control versus gefitinib). *right*, tumor volume in H1975 xenograft were measured on the last day of the experiment using vernier calipers. *Columns*, mean (n = 6); *bars*, SD. *P* = 0.0013 (control versus combination), 0.016 (control versus gefitinib) and 0.025 (gefitinib versus combination). The xenograft protocols were detail described in Materials and Methods.

To assess whether curcumin potentiates the antitumor activity of gefitinib *in vivo*, we examined the effects of curcumin and gefitinib on the tumor growth of the CL1-5, A549 and H1975 gefitinib-resistant cells via the xenografts model. We injected the cells subcutaneously into the mice; the mice were randomized and treated with vehicle control, curcumin (1 g/kg), gefitinib (120 mg/kg), and curcumin combination with gefitinib once daily orally after one week. Tumor volume from CL1-5 and H1975 xenografts mice were monitored per 4 days showing the similar results in the control group were gradual increased comparing with the other treatment groups (Figure 4A, left and 4B, left). The tumor volume in the combination group was significantly lower than the control group and showed the more inhibitory ability in tumor growth compared to the gefitinib versus vehicle control. Because the combined effect was the greatest in CL1-5 cell *in vitro*, we added one more treatment strategy in this xenograft model. The half-dose of the gefitinib (60 mg/kg) combined with curcumin showed the equal inhibitory effectiveness to the 120 mg/kg gefitinib alone group in the CL1-5 xenograft tumor volume. We monitored the tumor volumes on the last day of the experimental design indicated that the tumor volumes were statistically significant decreased in the combination groups compared with the vehicle control (60 mg/kg gefitinib combined with 1 g/kg curcumin *P* = 0.0006 versus control in CL1-5 xenograft; Figure 4A, right and 120 mg/kg gefitinib combined with 1 g/kg curcumin *P* = 0.0028 versus control in H1975 xenograft; Figure 4B, right). In addition, the effects on the combination groups versus control exhibited further growth inhibition in tumor volumes comparing to the gefitinib alone versus control in H1975 xenograft (*P* = 0.0028 and 0.02, respectively; Figure 4B, right). The results also indicated that the tumor volumes in 60 gefitinib combined with 1 g/kg curcumin group compare with 120 mg/kg gefitinib alone group in the CL1-5 xenograft was no significantly different (*P* = 0.484 versus gefitinib; Figure 4A, right).

We also evaluated the effects of curcumin and gefitinib in the growth of tumor volumes in A549 expressing luciferase xenograft model using IVIS imagining. Tumor volumes were monitored per 7 days and the bioluminescence imaging results (Figure 4C, left) indicated the significant lower in tumor volume of the gefitinib and combination group compared with the control group (*P* = 0.019 versus control). The results also showed that combination group significantly decreased the tumor volumes compared with the gefitinib alone to the vehicle control (*P* = 0.019 versus *P* = 0.146, respectively). The final tumor volumes on day 42 showed significant decrease in the combination group compared with vehicle control (*P* = 0.0013 versus control) and with gefitinib alone group (*P* = 0.025 versus gefitinib) (Figure 4C, right).

### Curcumin down-regulates EGFR, AKT, c-MET cyclin D1 and PCNA proteins expression, and induces apoptosis activation in CL1-5 xenograft tumors

**Figure 5:**
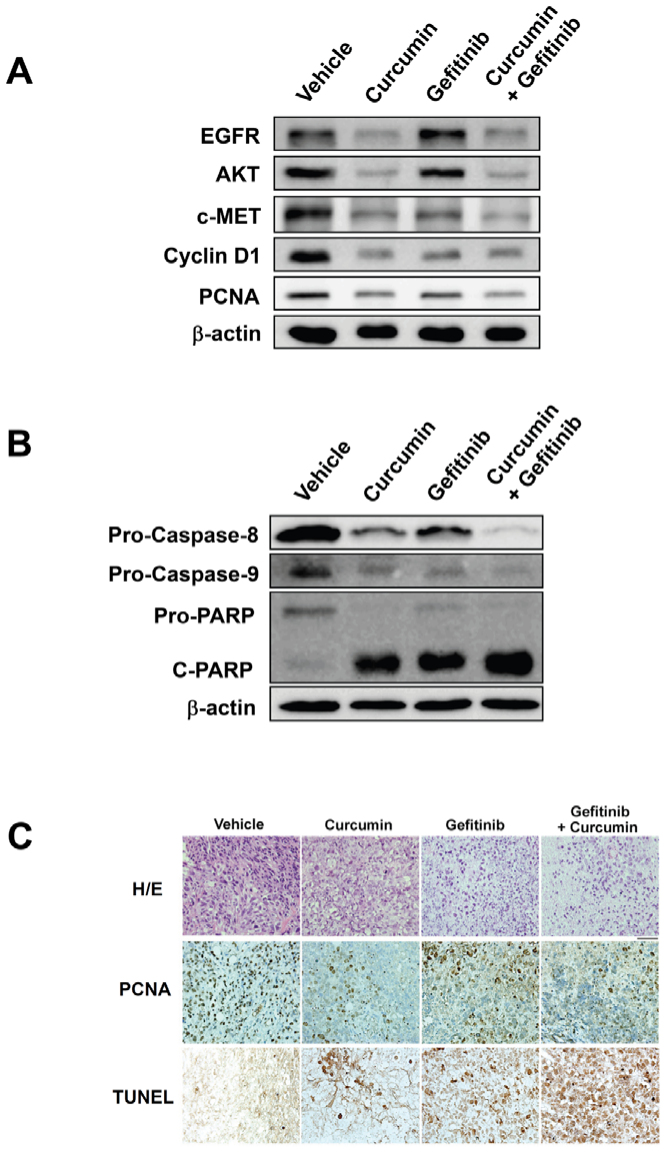
Curcumin enhances the effect of gefitinib against gefitinib-resistant relative proteins and by inducing apoptosis activity CL1-5 xenograft tumors. (A) Western blotting analysis the gefitinib-resistant relative proteins EGFR, AKT, c-MET, cyclin D1, and PCNA showed the expression inhibition in curcumin alone or in combination group of xenograft tumor tissues. Western blotting results are represented with β-actin used as the internal control. Samples were pooled together from three animals in each group were analyzed and representative data are shown. (B) Western blotting analysis showing that combination of curcumin and gefitinib inhibits the expression of pro-caspase-8, pro-caspase-9 and pro-PARP and enhances the level of the cleavage (c)-PARP in xenograft tumor tissues. Western blotting results are represented with β-actin used as the internal control. Samples were pooled together from three animals in each group were analyzed and representative data are shown. (C) H&E staining, immunohistochemical staining of proliferation marker PCNA and apoptosis detection of TUNEL analysis in xenograft tumor sections from each group indicating that curcumin inhibited the tumor cell proliferation and enhanced the apoptotic cell death of gefitinib under a light microscope (×400 magnification).

To examine whether curcumin potentiates antitumor effect of gefitinib on the tumor growth is associated with down-regulation of the tumor progression related oncogenic proteins expression in tumor tissues from four groups of CL1-5 xenograft. We tested the EGFR, AKT, c-MET cyclin D1 and PCNA proteins expression using Western blotting. The results showed the protein levels of EGFR and AKT were decreased in the tumor homogenates in both curcumin alone and curcumin combined with gefitinib groups, whereas, the c-MET, cyclin D1, and PCNA were also down-regulated simultaneously (Figure 5A).

To investigate whether curcumin sensitizes the apoptosis-inducing effects of gefitinib in CL1-5 xenograft tumors, the apoptosis-related protein caspase 8 and 9, and PARP were examined by Western blotting. The results showed that curcumin enhanced the activity levels of caspase-8, caspase-9 and PARP showing the decrease amounts of pro-caspase-8, pro-caspase-9, pro-PARP, and increasing the c-PARP, especially in the combination group (Figure 5B).

We next confirm the Western blotting results using immunohistochemical staining and cell apoptosis detection assay in CL1-5 xenograft tumor tissues. We examined that the PCNA-positive cells were decreased, which indicated the reduction of proliferated cells, in the curcumin treated and in the curcumin combined with gefitinib treated groups. In addition, the results showed that treatment with curcumin combined with gefitinib significantly increased cell apoptotic activities, TUNEL-positive cells, compared to the control and to the gefitinib groups (Figure 5C).

### Curcumin reduces the gefitinib-induced villi damage and apoptosis in mice intestines

**Figure 6:**
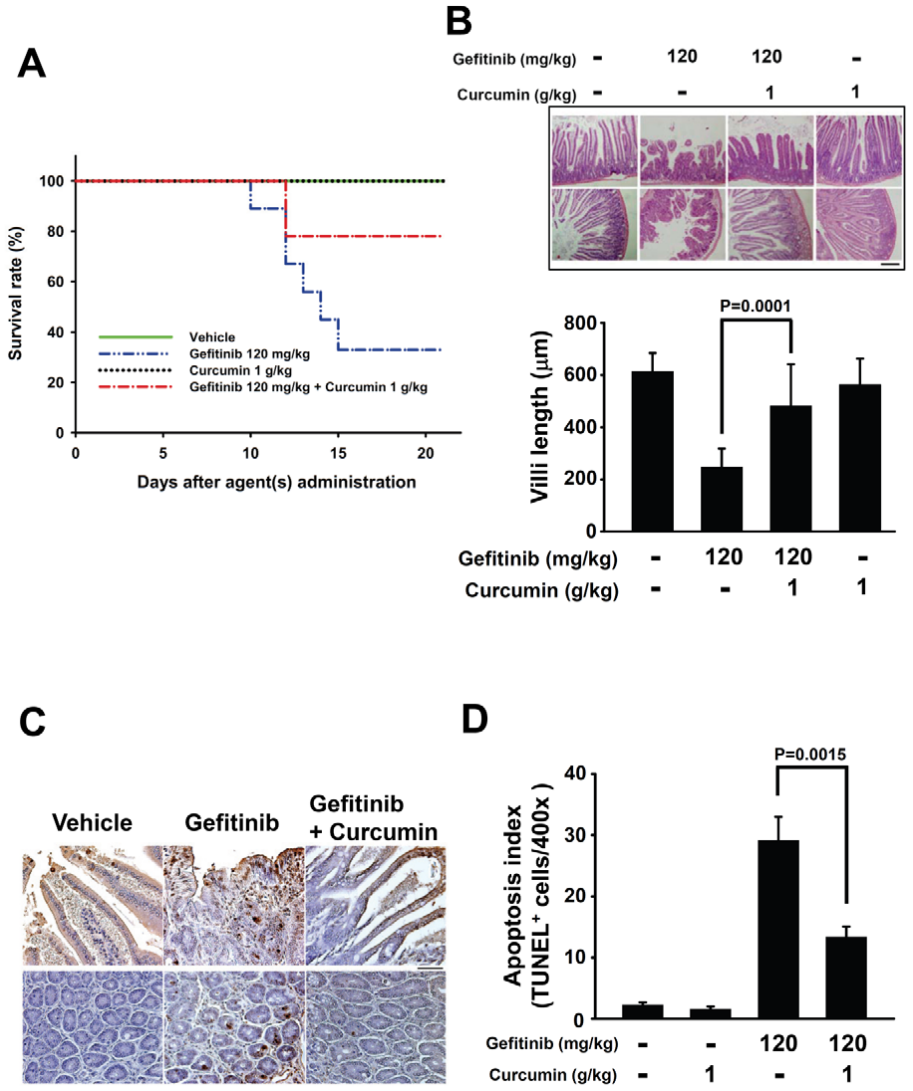
Curcumin attenuates the gastrointestinal adverse effects of gefitinib *in vivo*. (A) The animal survival rates for each group indicate that curcumin decreased mouse death caused by the adverse effects of gefitinib. (B) H&E staining analysis of intestine sections from each group showing that curcumin prevents villi damage; villi lengths were quantified (bar = 200 µm). *Columns*, mean (n = 3); *bars*, SEM. *P* = 0.0001 (gefitinib versus combination). (C) Apoptosis detection by TUNEL assay using intestine sections from each group. The results indicated that curcumin reduced the TUNEL-positive cells in the villi in the presence of gefitinib. (D) Quantification of TUNEL-positive cells as described in Materials and Methods. *Columns*, mean (n = 10); *bars*, SEM. *P* = 0.0015 (gefitinib versus combination).

During the *in vivo* xenograft study, we observed that the 120 mg/kg gefitinib-treated mice suffered the diarrhea side effect; this is similar to previous reports in clinical literature [28], [29] and was even severely to cause death within the group. We measured that the 22.17±0.18% body weight lost compared to the vehicle control group and there was also an obviously diarrhea side effect. The body weight didn't alter in the curcumin group as well as vehicle control. Interestingly, we found that the combination group showed to prevent the body weight loss at the last day of the study (13.19±0.15% loss comparing to the control groups) (data not shown) and significantly reduced the number of deaths among the mice (combination group was 78% compared with 33% for the gefitinib) (Figure 6A). We next examined the histological morphology in intestine tissues from four groups of the xenograft mice using H&E staining. The results in Figure 6B showed that the villus of the intestine tissues in the gefitinib group was shorter and looser than in the combination group. The length of villi in curcumin and gefitinib combined group were longer and had greater integrity compared to gefitinib group (*P* = 0.0001).

We next tested whether curcumin reduces the gefitinib-induced apoptosis effects in the mice villi from four groups. The results indicated that curcumin combined with gefitinib significantly reduced the expression of TUNEL-positive cells in intestine villi compared with the gefitinib group (*P* = 0.0015) (Figure 6C and D).

### Curcumin attenuates the adverse gastrointestinal effects of gefitinib by modulating p38 activation

**Figure 7:**
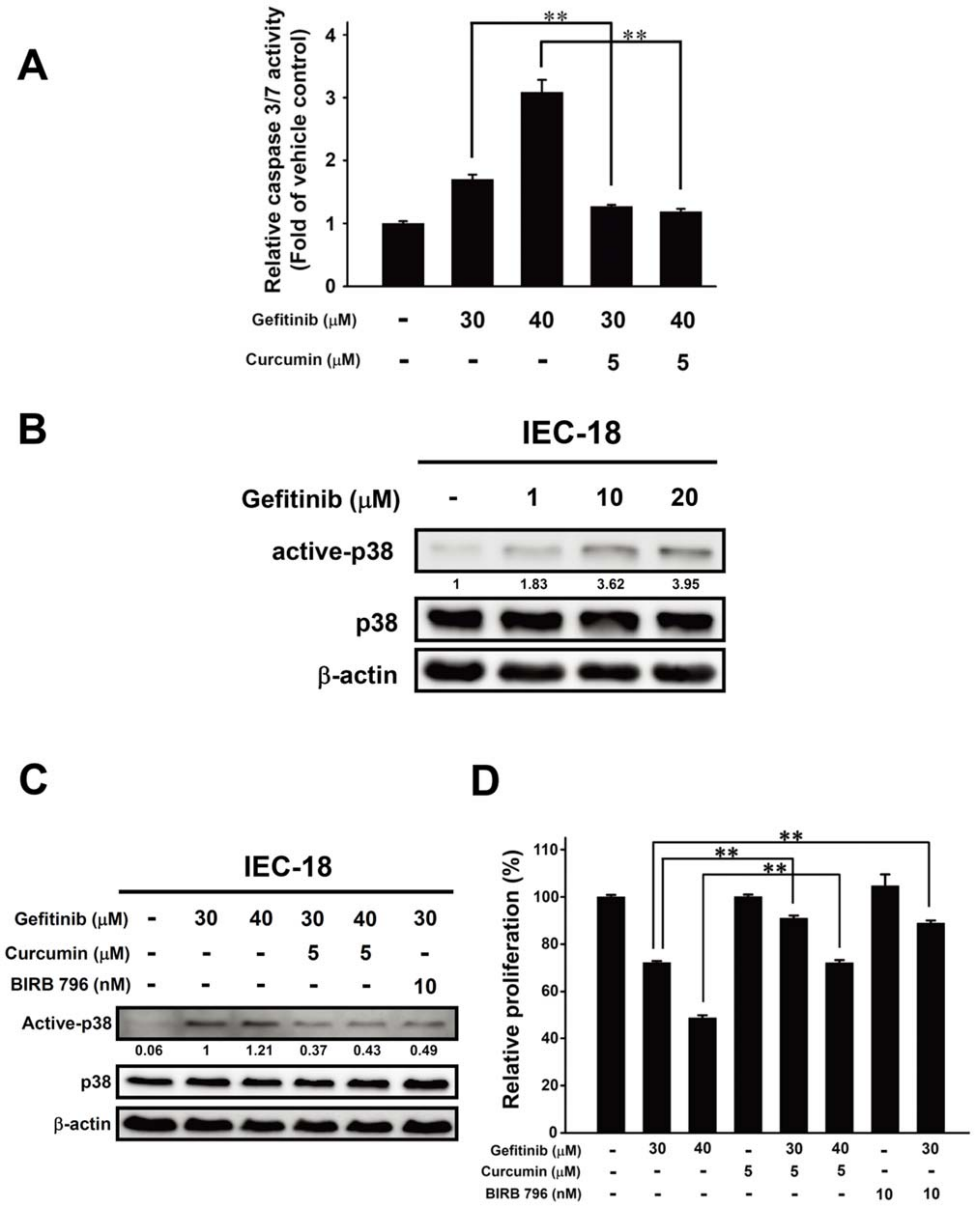
Curcumin reduces gefitinib-induced gastrointestinal damage through modulating p38 activation *in vitro*. (A) Caspase-Glo 3/7 assay analysis showing gefitinib induced caspase 3/7 activity in the dose-dependent manner in IEC-18 cell, but curcumin reversed this gefitinib-induced caspase activity. *Columns*, mean (n = 3); *bars*, SD. **, *P*<0.01. Data are representative of two independent experiments. (B) Western blotting analysis showed that gefitinib increased active-p38 expression in a dose-dependent manner (0–20 µM). β-actin used as the internal control and the numbers below the row of the active-p38 indicate the densitometric values normalized with the relative β-actin value. (C) Western blotting analysis showed that curcumin and BIRB 796 decreased the gefitinib-induced p38 activation protein level in IEC-18 cell. β-actin used as the internal control and the numbers below the row of the active-p38 indicate the densitometric values normalized with the relative β-actin value. (D) MTT assay results showed curcumin and BIRB 796 prevent the IEC-18 cell viability from the gefitinib-induced toxic damage. *Columns*, mean (n = 6); *bars*, SEM. **, *P*<0.01. Data are representative of two independent experiments.

To determine the possible mechanism of the effect of curcumin treatment on reducing gefitinib-induced damage, we used the non-transformed intestinal epithelial cell line IEC-18 to assess the protective effects of curcumin. We examined whether gefitinib might induce apoptosis in IEC-18 cell line measuring by caspase 3/7 activation (Caspase-Glo assay). The results showed that gefitinib (IC_25_ at 30 µM and IC_50_ at 40 µM of gefitinib to IEC-18 cell, respectively) induced caspase 3/7 activities in IEC-18 cell, whereas, 5 µM curcumin (non-toxic dosage) significantly inhibited the gefitinib-induced caspase 3/7 activities (Figure 7A). To study the possible mechanisms involve in this protective effect of curcumin on gefitinib-induced apoptosis, we found that the previous reports has indicated that gefitinib induced apoptosis in intestinal epithelial cells via p38 MAPK-dependent activation [30]. Herein, the results showed the active-p38 significantly increased in a dose dependent manner of gefitinib (0–20 µM) treatment in IEC-18 cell by Western blotting (Figure 7B), but not in the NSCLC cell lines (data not shown).

We next examined whether curcumin reduces the gefitinib-induced p38 activation and results in the better cell viability. The Western blotting results (Figure 7C) showed that curcumin (5 µM) significantly reduced the gefitinib (30 and 40 µM)-induced p38 activation in IEC-18 cell. The results also indicated that curcumin and the selective p38 MAPK inhibitor BIRB 796 (10 nM) decreased the active-p38 expression in gefitinib-treated IEC-18 cells. To examine whether curcumin sustains the viability in the presence of gefitinib *in vitro*, we assessed the MTT assay to determine the effect of curcumin on the gefitinib-treated IEC-18 cell. The results showed that gefitinib (30 and 40 µM) reduced IEC-18 cell survival, whereas, curcumin (5 µM), as well as the BIRB 796 (10 nM), significantly rescued the cells from this toxic effect of gefitinib (Figure 7D).

## Discussion

**Figure 8:**
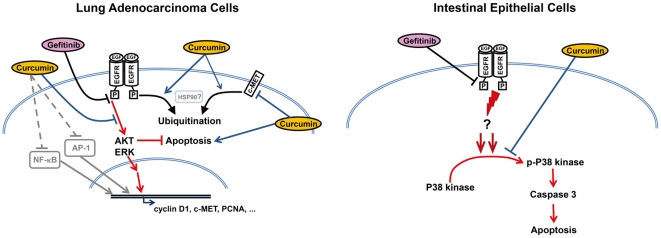
Scheme of the curcumin regulatory effects on lung adenocarcinoma cells and intestine epithelial cells. Curcumin enhances the antitumor effects of gefitinib on lung adenocarcinoma are hypothesized to occur through the induction of EGFR degradation, the blockage of EGFR activation, promoting apoptosis, inhibiting the expression of c-MET, cyclin D1 and PCNA. On the other hand, curcumin attenuates the gefitinib-induced damage on intestinal epithelial cells through modulating p38 kinase activation and cell apoptosis.

Curcumin has long been known as a potential therapeutic or preventive agent for several major human cancers [31], [32], by regulating multiple targets on tumor signaling pathways, such as protein kinases, transcriptional factors and apoptosis-related proteins [20], [33], [34], [35], [36], [37]. Also, since curcumin displayed synergistic effects of several chemotherapeutic drugs, it has been suggested as an adjuvant for anti-cancer therapy [36], [38], [39], [40], [41]. However, the potential anti-tumor effect of curcumin combined with EGFR-TKIs on NSCLC has not yet been investigated. In the present work, we found that curcumin has dual beneficial effects on gefitinib therapy in NSCLC (Figure 8). Curcumin can sensitize cancer cells to gefitinib therapy in NSCLC with different EGFR status through accelerating EGFR ubiquitination; also, curcumin can attenuate gefitinib-induced gastrointestinal adverse effects via altering p38 activation and reducing cell apoptosis in intestinal epithelial cells. We suggest curcumin could be clinically applicable as an adjuvant to increase the applications of EGFR-TKIs therapy in NSCLC patients with different EGFR status. This is the first report to show that curcumin can overcome the gefitinib inefficiency and inhibited EGFR protein kinase activity through down-regulating endogenous EGFR level via accelerating proteasome-ubiquitin activity. In addition, we showed that curcumin promoted gefitinib-induced cell apoptosis in the xenograft NSCLC tumor growth model via inducing caspase and PARP activation. The hypothetic scheme of curcumin enhanced anticancer effects of gefitinib on lung adenocarcinoma was shown in Figure 8.

Our results indicated that curcumin significantly reduced not only pEGFR, but also EGFR protein expression in a concentration-dependent manner. Although previous studies have indicated that curcumin inhibited EGFR kinase activity and block ligand-induced EGFR activation in the different types of cells [21], [22], these reports showed the amount of EGFR did not alter in the presence of curcumin. Our results found that curcumin can decrease EGFR protein expression might through down-regulated EGFR protein in gefitinib-resistant NSCLC cells. Even the MG132 treatment did not completely recovered the EGFR protein level in the presence of curcumin, we indicated that curcumin contributed to alter the EGFR stability and sensitized the NSCLC to gefitinib. Recently, the evidence indicated that curcumin can down-regulate EGFR protein expression as well as the function of Hsp90 inhibitor via the ubiquitin-proteasome pathway [42]. The molecular chaperone Hsp90 is a potential target for cancer treatment and regulates the client protein activation and stability such as self-sufficiency in growth signals, evasion of apoptosis, acquisition of limitless, insensitivity to antigrowth signals, invasion and metastasis and sustained angiogenesis [43]. Our data showed that curcumin down-regulated the EGFR expression in a dose-dependent manner were similar to the previous reports on the role played by Hsp90 inhibitor in the gefitinib-resistant NSCLC cell lines [44], [45], [46]. In addition, our results showed that Hsp90 client proteins: AKT, c-MET and cyclin D1 expressions were inhibited by curcumin in the xenografts model as well as the effect of Hsp90 inhibitor. Thus, these results suggested curcumin sensitized gefitinib and overcame the gefitinib resistance in modulating EGFR or other oncogenic proteins stability might similar to the actions of the Hsp90 inhibitor in which contributed to the NSCLC therapy (Figure 8).

Recent studies have described that cyclin D1 is a key driver of malignant transformation [47], and c-MET amplification leads to acquired TKIs resistance in NSCLC [48], [49]. Previous research exhibited that curcumin suppressed NF-κB-regulated proteins control cancer cell progression and these include cyclin D1 [33], [36] and blocked the transactivation of AP-1 on the MET promoter [50]. Our results showed that curcumin down-regulated cyclin D1 and c-MET expression in lung adenocarcinoma xenograft tissues. Thus, our results indicated curcumin may be an alternative therapeutic agent to combine with gefitinib for altering cyclin D1 and c-MET activation in acquired gefitinib-resistant NSCLC.

In the present study, we showed that curcumin combined with low dose gefitinib (1 µM) exhibited more anticancer activities as comparing with high dose of gefitinib (10–20 µM) treatment in inhibiting cell proliferation, decreasing colony formation ability and inducing apoptosis of these gefitinib-resistant lung adenocarcinoma cell lines *in vitro*. Besides, in CL1-5 xenograft mice model, half dose of gefitinib (60 mg/kg) plus curcumin group has similar anticancer activity in tumor growth to the 120 mg/kg gefitinib group. This is the first report indicates the potential of curcumin as an effective agent combine with gefitinib enhancing the antitumor activities, our data offer a potentially benefit for clinical application of curcumin in reducing the dosage of gefitinib. It might cut down the cost of targeted therapy drug gefitinib and abating the burden of patient and public health insurance. Due to the low absorption efficacy of curcumin, the dosage was used up to 1 g/kg. Although there is no toxic response observed during the whole experimental periods comparable to the previous study [36], this is still an issue for clinical application to exhibit sufficient beneficial effects of curcumin. Solving this problem, to innovate the novel preparation or analogs of curcumin for improving oral bioavailability was developed to prolong the half-life, reduce the dosage and improve the effectiveness of curcumin [51], [52], [53]. From the herbal and natural compounds screening to the evaluation of the effects on the hit of curcumin in NSCLC in our study, we suggests that curcumin might be an ideal leading compound for drug development or as an adjuvant in the NSCLC treatment.

The evidence in previous xenografts study indicated that at the maximum tolerated dose (150 mg/kg) of gefitinib showing 70–80% growth inhibition again A549 tumor and the body weight was 10% after qd × 5 for two weeks treatment in mice [54]. In the present study, 120 mg/kg gefitinib was used as the high dose treatment and 60 mg/kg as the lower dose to test the effects on tumor growth in the alone or combined with curcumin treatment in gefitinib-resistant cell lines xenograft. Although the results indicated the tumors growth inhibition were different in the three gefitinib-resistant NSCLC cell lines xenografts after gefitinib alone treatment; whereas, combination with curcumin showed the significant regression in tumor growth compared with gefitinib alone.

In the present work, the high dose of gefitinib (120 mg/kg) caused the reduction of the survival rate in mice after the third week treatment and the body weight loss were significantly and observably severe diarrhea compared to the other groups. Previous reports have shown that treatment with gefitinib always leads to diarrheal side effects in patients [28], [29], which opens up to question as to what is the possible mechanism of the GI side effects of gefitinib and how combination treatment with curcumin can rescue the survival rate? Recent studies indicated that diminish EGFR activity initiates the intrinsic pathway of cell apoptosis accompanied by rapid phosphorylation of p38 and Bax activation in intestinal epithelial cells. [30], but gefitinib did not influence the expression or phosphorylation state of p38 in NSCLC *in vitro* and *in vivo* [55], [56]. In addition, curcumin has been discovered as a p38 modulator [57], [58]. These studies may provide the possible hint for us to solve the gefitinib-related adverse effect problem in the GI. The hypothetic scheme of curcumin attenuated the gefitinib-induced apoptosis on intestinal epithelial cells in this study was shown in Figure 8. Our result is the first report to show that curcumin significantly attenuated the gefitinib-induced diarrheal side effect, prevented the loss of integrity of the intestine villi and reduced apoptosis in the intestine *in vivo*. In addition, the intestinal epithelial cells protective effects of curcumin can be validated *in vitro* in the IEC-18 rat intestinal epithelial cell line by reducing the gefitinib-induced apoptosis and preserve cell viability in the presence of gefitinib might through modulating p38 activation, comparable to other previous findings showed that curcumin can protect intestinal mucosa barrier and other related intestinal diseases through inhibition of p38 and NF-κB activation, modulating IL-10, IL-β, and MMP-3, and other anti-inflammatory and anti-oxidant activities [59], [60].

In conclusion, our novel findings suggest that curcumin, a natural food substance with no known human toxicity, holds promise as an adjuvant to broaden the spectrum of gefitinib therapy to improve the efficacy of gefitinib among the NSCLC patients with different EGFR status. Our finding is the first report showing curcumin is able to exhibit the prominent activity which enhances anti-tumor abilities of gefitinib *in vitro* and *in vivo* by modulating EGFR stability. Curcumin also attenuates gefitinib-induced GI damage by modulating p38 activation. To overcome the gefitinib inefficiency and to prevent the gefitinib-induced diarrhea side effect, we suggest that curcumin might be an ideal adjuvant for NSCLC patients during gefitinib treatment. Further clinical studies are needed to prove our findings of curcumin in NSCLC patients.

## Materials and Methods

### Ethics statement

The *in vivo* study protocols were approved by the Institutional Animal Care and Use Committee of the College of Medicine, National Taiwan University (approval number IACUC-20090083). All procedures were performed under minimize suffering.

### Cell lines

The human lung adenocarcinoma cell line CL1-5 was established previously [61]. A549, H1299, H1650, H1975 (table 1), and the human bronchial epithelium cell line BEAS-2B were obtained from American Type Culture Collection (ATCC) (Manassas, VA). PC-9 was a kindly gift from Dr. Chih-Hsin Yang (National Taiwan University Hospital, Taiwan). These cells were grown in RPMI 1640 medium. The IEC-18 rat intestinal epithelial cell line (ATCC, CRL-1589™) was obtained from Bioresource Collection and Research Center (BCRC, Taiwan) grown in DMEM medium. The media of the above contained 10% FBS (Life Technologies) with penicillin and streptomycin (100 mg/ml) were incubated at 37°C in a humidified atmosphere of 5% CO2.

**Table 1:** The EGFR status and ethnicities of the NSCLC cell lines used for herb compounds screening.

Cell line	*EGFR* status	Ethnicity
CL1-5	wild-type *EGFR*	Asian
A549	wild-type *EGFR*	Caucasian
H1299	with wild-type *EGFR* but p53-null	Caucasian
H1650	in-frame deletions (ΔE746-A750) of exon 19 in *EGFR,* but loss of PTEN protein	Caucasian
H1975	L858R substitution in exon 21 and secondary T790M substitution in exon 20 in *EGFR*	Caucasian
PC-9	in-frame deletions (ΔE746-A750) of exon 19 in *EGFR*	Asian

### Drug treatment

Curcumin was purchased from Sigma (St Louis, MO). Gefitinib (ZD1839) was kindly provided by Astra-Zeneca Pharmaceuticals (Macclesfield, UK). Stock solutions of curcumin and gefitinib were prepared in dimethyl sulfoxide (DMSO) and stored at −20°C. The compounds were diluted in fresh media before each experiment, and the final DMSO concentration was lower than 0.1%. Cells were plated in 10-cm dish at a density of 1×10^6^. After incubating overnight, cells were serum starved for 24 h without FBS. Cells were treated with 1–20 µM curcumin and/or gefitinib for 1 h in a serum-free condition and then were stimulated with 20 ng/mL EGF (Sigma, MO) for 30 min. For the detection of the time course in EGFR degradation, CL1-5 cells were plated in 10-cm dish at a density of 1×10^6^ for 24 h starvation. Cells were treated with 100 µg/ml cycloheximide and 10 µM curcumin for 1 h and then added 20 ng/ml EGF. Cells were collected at 0–60 min as indicated. For the immunoprecipitation, cells were plated in 10-cm dish at a density of 1×10^6^ for 24 h seeding. 10 µM MG132 (Sigma, MO) was added for 1 h and then treated with 5–20 µM curcumin for further 1.5 h. For the IEC-18, the cells were treated with gefitinib and/or curcumin or BIRB 796 for 24 h. Cells were washed thrice with ice-cold PBS and collected for protein extraction.

### High-throughput drug screening

The herbal and natural compounds library includes the collection of the 598 pure products and their derivatives purchased from Sigma (St Louis, MO) and ChromaDex (Irvine, CA), containing a range of alkaloids, sesquiterpenes, diterpenes, pentacyclic triterpenes, sterols, and many other diverse representatives. CL1-5, A549, H1299, H1650 and H1975 cells were used for screening by the proliferation assay; the PC9 as gefitinib-sensitive control. For exclude the toxicity to normal cells, BEAS-2B was used as the normal cell control.

### Proliferation assay

MTT [3-(4,5-dimethylthiazolyl-2)-2,5-diphenyltetrazolium bromide] (Sigma, St Louis, MO) assay was performed to determine cell proliferation. Briefly, CL1-5, A549, PC-9, H1650, H1975 and IEC-18 cells were plated in 96-well plates at a density of 5×10^3^ cells/well. After incubating for 24 h, cells were treated with different concentrations of curcumin and/or gefitinib for 72 h. The IEC-18 cells were treated with curcumin, BIRB 796 and/or gefitinib for 24 h incubation. 0.5 mg/ml MTT solution in the culture medium was then added to the wells. After a further 1.5 h of incubation, the medium was removed and DMSO was added to the plates. The color intensity was measured at 570 nm using a multi-label plate reader (Vector3; Perkin-Elmer, USA).

### Colony formation assay

CL1-5, A549 and H1975 cells were plated in 6-well plates (100 cells per well). After incubating for 24 h, the cells were treated with 1–15 µM gefitinib or 1–15 µM curcumin alone or with a combined treatment. The cells were cultured with the agents for 5 days and then changed in the complete culture medium; the cells were incubated for a further 9 days. Colonies were stained using 0.001% crystal violate and the number of colonies per well were counted.

### Western blot analysis

Cells and xenograft tumor tissues (100 mg) lysates were prepared in mammalian protein extraction reagent (Pierce, Rockford) containing protease inhibitor and phosphatase inhibitor (Sigma, MO) and clarified by centrifugation. Protein concentrations were determined by the BCA assay (Pierce, Rockford). SDS/PAGE with 10% resolving gel was used to separate proteins (40 µg/lane). Phospho-EGFR (Tyr1068), phospho-AKT (Ser473), AKT, c-MET, caspase-3, caspase-8, caspase-9 and PARP antibodies were purchased from Cell Signaling Technology (Beverly, MA). Total EGFR, cyclin D1 and PCNA were purchased from Santa Cruz Biotechnology (Santa Cruz, CA). Antibodies were used according to the conditions recommended by the manufacturer. Bound antibody was detected using the Enhanced Chemiluminescence System (Santa Cruz, CA). Chemiluminescent signals were captured using the Fujifilm LAS 3000 system (Fujifilm, Tokyo, Japan).

### Immunoprecipitation

For the detection of EGFR-ubiquitin complex, whole-cell lysates were obtained in IP lysis buffer [50 mmol/l Tris (pH 7.4), 150 mmol/l sodium chloride, 0.1% NP40] containing protease inhibitor and phosphatase inhibitor (Sigma, MO) and clarified by centrifugation. Protein concentrations were determined by the BCA assay (Pierce, Rockford). Cell lysates (1 mg) were incubated overnight with the EGFR IP-specific mouse monoclonal antibody (Cell Signaling, MA) at 4°C. Protein A-Sepharose beads (Santa Cruz, CA) were added to the immunoprecipitates and incubated at 4°C for 1 h. The beads were collected by centrifugation at 1,000 g for 2 min and washed 4 times in PBS with protease inhibitor and phosphatase inhibitor. Proteins were eluted with the SDS protein sample buffer before the Western blotting analysis with specific antibodies against ubiquitin and EGFR (Santa Cruz, CA).

### Synergy

The results from the proliferation assays were entered into the CalcuSyn software (Biosoft, Cambridge, UK) to determine the presence of synergy between curcumin and gefitinib in five NSCLC cells. Combination index (CI) isobologram was calculated in an algebraic estimate according to the method described by Chou and Talalay [62]. CI <1, CI = 1 or CI >1 represent synergism, additive effect or antagonism of both compounds, respectively.

### Flow cytometry

CL1-5, A549 and H1975 cells were seeded in 6-mm culture plate at the density of 1×10^5^ cells per dish. After incubating for 24 h, cells were treated with 1 and 20 µM gefitinib with/without 15 µM curcumin for further 72 h. Adherent and floating cells were both collected and resuspended in cold PBS for analysis. Cells were stained with Annexin V-FITC Apoptosis Kit (BD Pharmingen, USA) to monitor apoptosis cells and propidium iodide (PI) to detect dead cells. Samples were analyzed on the FC 500 Flow Cytometry Systems (Beckman Coulter).

### In vivo study protocol

The CL1-5, A549 and H1975 cells were calculated the cell viability and cell number by trypan blue, finally 1×10^6^ live cells in 100 µl HBSS were injected subcutaneously into 6-weeks-old SCID mice (supplied by the animal center in the College of Medicine, National Taiwan University, Taipei, Taiwan). A549 cells were stably transfected with luciferase was a kindly gift from Professor Win-Ping Deng (Taipei Medical University, Taiwan). Groups randomized and treatment started when tumor size was 3–5 mm. The A549 xenograft mice were randomized based on the first bioluminescence measured after the IVIS imaging (IVIS Imaging System 200; Xenogen Corp., Alameda, CA) were used according to the conditions recommended by the manufacturer. Each experimental group included at least 6 mice bearing tumors. Curcumin for animal was prepared and suspended properly in propylene glycol (J.T.Baker, Phillipsburg, NJ) and gefitinib was prepared in drinking water. The dosage of 1 g/kg curcumin alone without toxic effects to mice was used as descript in previous study [36] and gefitinib alone (120 mg/kg) or the combination of curcumin and gefitinib were fed through oral administration and once daily at indicated dose for treatment. Tumor sizes of CL1-5 and H1975 were monitored per 4 days by electronic vernier caliper and the tumor volume was calculated by using the formula *V* = 0.4×*ab* ^2^, where *a* and *b* are the longest and shortest diameters of the tumors, respectively. The photons volume of A549 bearing tumors were monitored per 7 days by the IVIS imaging. The mice were sacrificed and subcutaneous tumors were excised and half of the samples were frozen in liquid nitrogen and stored at −80°C after several weeks of the proper end point. Half of the tumor tissues were formalin fixed and paraffin embedded for immunohistochemistry and routine hematoxylin and eosin staining.

### Immunohistochemical staining and cell apoptosis detection assay

Cell proliferation analysis was performed on the paraffin-embedded tumor tissue samples using PCNA staining. Briefly, a rabbit anti-human PCNA polyclonal antibody (Santa Cruz, CA) was used in the primary reaction. The DAKO EnVision System, containing a secondary horseradish peroxidase-conjugated anti-rabbit antibody complex, was used with 3,3′-diaminobenzidine to detect the PCNA.

Colorimetric immunohistochemical staining for apoptotic cell death (TUNEL) was performed on the paraffin-embedded tumor and intestine tissue sections using the *In Situ* Cell Death Detection Kit, POD (Roche Diagnostics, Germany); the sections were also counterstained with Gill's hematoxylin. TUNEL-positive cells were examined in 10 random fields from 4 treatment groups and then expressed as the mean number of TUNEL-positive cells ± SEM per high-power field (×400 magnification).

### Measurement of caspase activity

Caspase activity was detected by using Caspase-Glo 3/7 assay kit (Promega Corporation, Australia). Briefly, The IEC-18 cell was seeded in 96-well white luminometer assay plates at a density of 1×10^4^ cells per well and incubated at 37°C. Cells were treated with gefitinib and/or curcumin for 24 h. 100 µl caspase 3/7 reagents were added to each well and incubated for 1 h on rotary shaker at room temperature. The luminescence intensity was measured using a multi-label plate reader (Vector3; Perkin-Elmer, USA).

### Statistical analysis

Statistical analysis was performed using two-tailed Student's *t* test and significant difference was defined as *P* <0.05. Data are presented as mean ± SD or SEM.
